# Visual information and expert’s idea in Hurst index estimation of the fractional Brownian motion using a diffusion type approximation

**DOI:** 10.1038/srep42482

**Published:** 2017-02-14

**Authors:** Ali R. Taheriyoun, Meisam Moghimbeygi

**Affiliations:** 1 Shahid Beheshti University, G.C., Department of Statistics, Tehran, 1983969411, Iran

## Abstract

An approximation of the fractional Brownian motion based on the Ornstein-Uhlenbeck process is used to obtain an asymptotic likelihood function. Two estimators of the Hurst index are then presented in the likelihood approach. The first estimator is produced according to the observed values of the sample path; while the second one employs the likelihood function of the incremental process. We also employ visual roughness of realization to restrict the parameter space and to obtain prior information in Bayesian approach. The methods are then compared with three contemporary estimators and an experimental data set is studied.

## General motivation

Fractional Brownian motion (fBm) appears in modeling wide classes of non-stationary stochastic processes. The statistical self-similarity and the ability of to fine-tune the order of Hölder continuity are the famous advantages of this process that make it typically one of the greatest interest in modeling natural phenomena. Typically, the creation of the fBm is attributed to ref. 1 since it investigated the basic properties of fBm and stressed its role in modeling of natural phenomena. However, it had been introduced during the generation of Gaussian spirals in Hilbert space^2^. Let 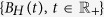 be an fBm that is a Gaussian zero-mean continuous process with stationary increments and the homogeneous stationary incremental variance function


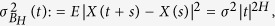


for any *t, s* > 0. Consequently, the covariance function is


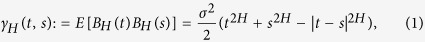


where 0 < *H* < 1, and *σ* > 0 are the parameters of the process. *σ* is a scale parameter and we then assume that *σ* = 1. Therefore, the interesting parameter is *H*, called fractal or Hurst (by Benoit B. Mandelbrot) index, in honor of Harold E. Hurst (for spending 62 years in Egypt carrying out a project on the hydrology of the Nile river) and also in honor of Ludwig O. Hölder.

The features of *H* are more far-reaching than a parameter of covariance function. For instance, we know that the differentiability of a process indicates the smoothness of the corresponding realizations. According to the last term of (1), fBm is not differentiable in 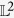, while it satisfies the Hölder continuity of order *H* and it has derivatives of order *α* for any 0 < *α* ≤ *H*. Thus, an fBm with greater *H* induces the smoother realizations and this is our general theoretic motivation for considering *H* as the crucial parameter of the process. Incremental stationary Gaussian processes with a Hölder continuity of order *H* construct an important class of *H*-index processes^3^. The most important feature of this family is the closed form of Hausdorff or fractal dimension for the graph of Gaussian processes of this family. More clearly, the Hausdorff dimension of an *H*-index process is equal to 2 − *H*. This means that the dimension and consequently the roughness of the fBm is decreasing in *H* (which is why *H* is called the fractal index) and stresses the importance of the estimation of *H*.

A very simple approach to obtaining the ML estimate is considering the likelihood function as the density function of a multivariate normal distribution. According to the last term of the covariance function (1), the joint density function behaves very roughly and is not hoped to employ even numerical methods to obtain the ML estimate of *H*. For a given index *t*, we simply use the series decomposition^4^ *B*_*H*_(*t*) = ∑_*n*_*Y*_*n*_(*t*), such that for each *n* the summand process {*Y*_*n*_(*t*)}_*t*_ has an Itô integral representation. The choice of *Y*_*n*_ depends on the true value of *H*. When *H* < 1/2, the summand process is denoted by *Y*_1*n*_ and for the case *H* > 1/2, the summand is in the form of *Y*_*n*_ = *Y*_2*n*_ + *Y*_3*n*_ where *Y*_*jn*_, *j* = 1, 2 and 3, is defined in the section entitled Decomposition of fBm. We then follow two scenarios in the ML computation: The first one is the simple use of the more computationally compatible covariance functions of the independent processes {*Y*_*n*_(*t*)}, *n* = 1, 2, … and then using the ML estimate of the covariances of *Y*_*n*_ and the invariance property of ML estimates, we compute the ML estimate of *H*. The second scenario is based on the covariance function of the incremental process *Y*_*n*_(*t*_*i*+1_) − *Y*_*n*_(*t*_*i*_) that is more smooth in comparison with fBm. Again, using the independence of *Y*_*n*_ and *Y*_*n*′_ for each 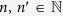, the likelihood function becomes more comfortable to apply numerical maximization algorithms. We hope also to accelerate the Bayesian computation by the use of this approximation of the likelihood function. The MCMC algorithm is used to capture the posterior information from the multiplication of the approximation of likelihood function by a prior distribution. Meanwhile, the visual information of the observed path of fBm is transferred to the estimation procedure by use of informative prior distributions. For instance, when the sample path shows an obvious short range dependence property, we consider the interval (0, 1/2] as the support of a prior distribution.

## Practical motivation

We discussed above the theoretical motivation for estimating *H* and now we briefly explain the enthusiasm of physicists and environmental scientists for *H*. Measuring the smoothness is an inseparable part of almost all surface analysis investigations. Due to the *power* and *scaling* laws, a variety of high resolution observations has been studied as a realization of fractal random fields. The Lévy fractional Brownian sheet (LfBs) of fractal index *H* is usually appointed to represent fractal behavior. The zero-mean Gaussian random field *L*_*H*_(**t**) is an LfBs of index *H* if


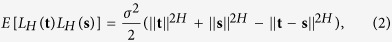


for 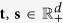. There are many methods to estimate the fractal index *H* based on the *d*-dimensional data of realization, but using almost all of them requires both computational and technical skills. A discussion on this subject could be found in ref. 5 and the references therein. Using the line transect data, we hope to decrease the computational cost of estimating *H* by reducing the dimension of data which tends to estimating *H* based on one-dimensional processes. For clarity, suppose that a picture has been taken of a surface (*d* = 2) and the observation is gathered in an *r* × *c* matrix. We are looking for an estimation of *H* that uses the data as *c* samples of length *r*. The question is: ‘is it possible to consider the data matrix as *c* sample paths from an fBm of index *H* ? ’ To answer this question, note that the Hausdorff dimension of the graph of *L*_*H*_(**t**) is equal to *d* + 1 − *H*. Extract the simple one dimensional stochastic process 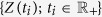 from *L*_*H*_(**t**), **t** = (*t*_1_, …, *t*_*d*_), by letting the *d* − 1 number of the elements of 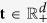 to be fixed. Let 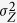 be the incremental variance of *Z*, then


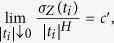


where *c*′ > 0 does not depend on **t**, and hence *Z* is a one-dimensional index-*H* process^3^. Thereupon, it is helpful to look at the line-transect data of the realizations of LfBs when the aim of the study involves only the estimation of the fractal index and/or measuring surface roughness.

The estimators of *H* have often been made for estimating the roughness of surfaces using the line transect data. Several methods have been proposed to estimate *H*, the oldest among them is R/S-statistic introduced by ref. 6. Further results for this estimator were provided by ref. 7. In ref. 8 a robust estimation of *H* has been introduced for stable distributions. It has been shown that this estimator is not efficient in comparison with the ML estimators in the Gaussian case. Perhaps the first serious attempt to make the ML estimate in image textures is provided by ref. 9. One may find a Bayesian solution for the same problem in ref. 10. Using the asymptotic behavior of the *k*-th absolute moment of discrete variations of a sampled path, a class of consistent estimators of the Hurst index has been constructed^11^. Based on the Bahadur representation for sample quantiles of nonlinear transformations of Gaussian processes, another consistent estimator of the Hurst index for the general class of locally self-similar Gaussian processes has been presented^12^. Generally, there is a class of estimators based on a method, called discrete variations, in which the oscillation of a quantity is employed in the estimation procedure. In statistical literature, refs 13 and 14, simultaneously originated this method and it has been highlighted many times in the other works^11^,^12^,^15^. There also exists a consistent estimator of *H*, based on the Karhunen-Loève expansion of a Gaussian process^16^. We compare our estimators with the estimators of refs 11, 12 and 15.

## Methods

### Decomposition of fBm

A stochastic integral representation of *B*_*H*_ has been introduced^17^ as follows:


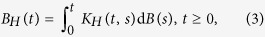


where {*B*(*t*), *t* ≥ 0} is the standard Brownian motion and


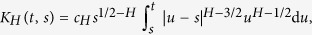


when 1 > *H* > 1/2 and


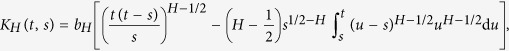


for 0 < *H* ≤ 1/2 where


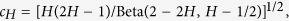



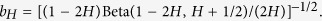


The mentioned representation in (3) induces the idea of achieving a diffusion process based on the stochastic integration. Since the solution of an Itô integral is a semi-martingale and since *B*_*H*_, as the solution of the stochastic integral in (3), is not a semi-martingale for *H* ≠ 1/2, the idea of constructing an exact diffusion representation is canceled. On the other hand, there is a series of diffusion processes, 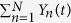, such that it converges^4^ to *B*_*H*_(*t*). The process {*Y*_*n*_(*t*)} is the answer of an Itô integral and strongly depends on the value of *H*. We would like to compute the likelihood function using the joint distribution of the diffusion processes {*Y*_*n*_(*t*)}_*t*_ but for finite *N*. Precisely, the series decomposition for *B*_*H*_(*t*) is obtained from the following convergences:


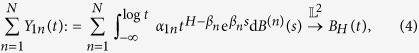


for 0 < *H* ≤ 1/2 and


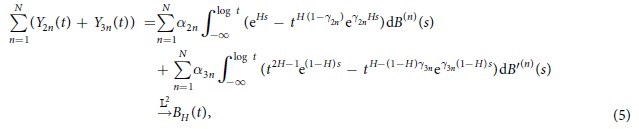


for 1/2 < *H* < 1, as *N* → ∞ where *B*^(*n*)^ and *B*′^(*n*)^ are independent sequences of independent Brownian motions. Also,


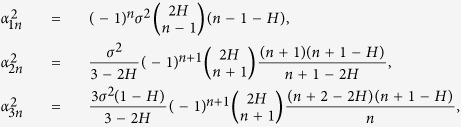


where 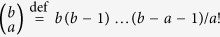 and *β*_*n*_ = |*n* − *H* − 1|. Clearly, for given *t* ∈ (0, 1) and *n* ∈ {1, …, *N*}, *Y*_2*n*_(*t*) and *Y*_3*n*_(*t*) are defined as


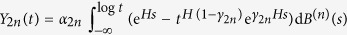



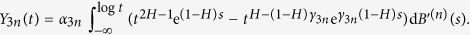


Based on the mentioned convergences in (4) and (5), we would like to construct the likelihood function by applying the Euler discretization on the diffusion processes. In the sequel denote the global parameter space (0, 1) by Θ and denote the restricted parameter spaces (0, 1/2] and (1/2, 1) by Θ_1_ and Θ_2_, respectively. One may suggest simpler approximations^18^ where the fBm is approximated using a semi-martingale process but the approximation does not have a diffusion-type representation. The presented approach strongly depends on this representation and therefore substitution of the approximation mentioned by ref. 4 with other approximations may lack the procedure of likelihood computation.

### Estimation of *H*: simple use of convergences

Assume that a sample path of *B*_*H*_(*t*) is observed at discrete times *t*_*i*_ ∈ [0, 1) for *i* = 1, …, *m*, where *m* is not a prime number. The restriction of time index into the interval *t* ∈ [0, 1) is somehow a loss of generality. But, using the self-similarity of the fBm, it is possible to rescale the sample path into this interval. In fact, this assumption is important in computing the variance of *Y*_*jn*_, for *j* = 1, 2 and 3. Moreover, only for this subsection let *m* = *kp*, where 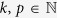. A very elementary approach is to consider the *B*_*H*_(*t*_*i*_) as a summation of independent Gaussian random variables.

**Proposition 1.** Let *λ*_*in*_(*t, s*) denotes *cov*(*Y*_*in*_(*t*), *Y*_*in*_(*s*)) for *i* = 1, 2, 3, and *n* = 1, …, *N*. For the *fBm, B*_*H*_, the covariance between two arbitrary points 0 < *s* < *t* satisfies


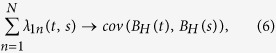


uniformly on *t* and *s* as *N* → ∞ when *H* ∈ Θ_1_. The convergence for *H* ∈ Θ_2_ is


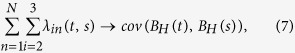


where


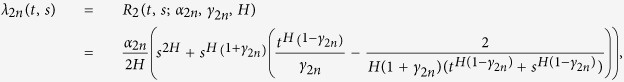


and


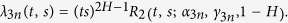


The proof of this proposition is grouped with all the other proofs in the supplementary file.

For a large enough *N*, the left-hand sides of (6) and (7) return an approximation of the covariance function of the fBm in terms of the mentioned series decomposition. We now consider the sample path as a dependent sample of size *k*, from a *p*-variate zero-mean normal random vector with covariance matrix ∑ = ∑_*n*_[*λ*_*ln*_(*t*_*i*_, *t*_*j*_)]_*ij*_, for appropriate *l* = 1, 2, where the elements are computed with respect to Proposition 1. There are various methods to compute the ML estimation of *H*, called 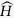, based on the approximation of likelihood. Only for this subsection assume that the sample path is observed regularly on *t*_*i*_ = *i*/*m*. The first method begins with the ML estimator of the autocovariance of the incremental stationary process with observations Δ*B*_*H*_(*i*/*m*) = *B*_*H*_((*i* + 1)/*m*) − *B*_*H*_(*i*/*m*) for *i* = 1, …, *m* − 1. Then, we compute the ML estimate of the covariance matrix of the *p*-dimensional random vector (Δ*B*_*H*_(*i*/*m*), …, Δ*B*_*H*_((*i* + *p* − 1)/*m*)) where the estimated matrix does not depend on *i*. This covariance matrix consists of *p* estimated autocovariances at lags 0, 1/*m*, …, (*p* − 1)/*m*. Using the invariance property of ML estimators one may recover 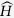 from each component of this matrix. The resulted estimates depend on the selected lag to recover the *H*; thus, based on this observation we may have *p* different values for the ML estimate of *H*. Simply speaking, if 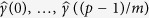 is the estimated autocovariances at the mentioned lags, then the 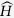
’s are the solutions of nonlinear equations


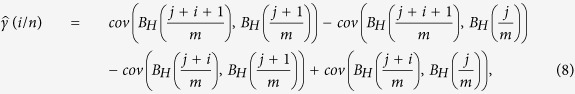


for *i* = 0, …, *p* − 1 and the equations do not depend on *j*. The covariances are replaced from the left hand side of (6) or (7) when *H* ∈ Θ_1_ or *H* ∈ Θ_2_, respectively. This equation produces *p* different values for the ML estimate of *H*, denoted by 
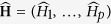
, and one may use the average of all the resulting solutions of (8) for various values of *i* to obtain a new consistent estimator. To this end, we propose the estimator


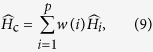


where *w* is an appropriate weight function. The idea of using the weighted average comes from the numerical properties of 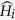. Numerically, those 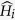
’s that correspond to the larger values of *i* return better estimates for *H*. It is worth mentioning that, although the larger values of *H* eventuate smoother realizations for the fBm, the error of approximation becomes larger for *H* > 1/2. Hence, the conjecture of better results for smoother realizations may not be true which is due to the weakness of convergence when *H* > 1/2.

### Estimation of *H*: Incremental approach

The approximation of the covariance function of fBm induces the idea of maximizing the likelihood function with respect to *H*, directly. The new problem is the complicated form of the inverse and determinant of the approximated covariance matrix. Since the process of increments of fBm is stationary, we expect more explicit form of the relationship between *H* and the covariances for the incremental process. The increments of process 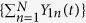 do not constitute a stationary process; however, for large enough *N*, they converge in 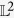 to the stationary incremental process of fBm. Using the second approach, we see that the appearance of *H* in the likelihood becomes more suitable via the smoothing effect of difference operator. This may accelerate the numerical methods of the maximization of likelihood.

Let **B**_*H*_ denotes the vector of sample path (*B*_*H*_(*t*_1_), …, *B*_*H*_(*t*_*m*_))^T^. Also, let 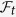 be the standard filter generated by the Brownian motion that is the smallest *σ*-field induced by {*B*^(*n*)^(*s*), *s* ≤ *t*}. We attempt to figure out the finite-dimensional distribution of increments Δ*B*_*H*_(*t*_*i*_) = *B*_*H*_(*t*_*i*+1_) − *B*_*H*_(*t*_*i*_), for *i* = 1, …, *m* − 1 and hence the likelihood function. The random variables *Y*_1*n*_(*t*_*i*_) and *Y*_1*n*_(*t*_*i*+1_) are 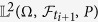, for 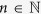 and {*B*_*H*_(*t*)} is a quadratic variation process. Thus, we get the convergence


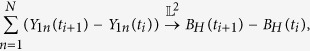


as *N* → ∞ for 0 < *H* ≤ 1/2. Convergence in 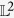 implies the convergence in distribution and we then look at the increments as the limit of the bi-indexed stochastic process 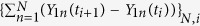. The following Theorem helps us to approximate the distribution of the increments of a sample path of fBm with Hurst index *H* ≤ 1/2.

**Theorem 1.** Suppose that **B**_*H*_ is a sample path of an *f**B**m* with fractal index *H* ∈ Θ_1_. The incremental process {Δ*B*_*H*_(*t*_*i*_), *i* = 1, …, *m* − 1} is the limit of a bi-indexed zero-mean Gaussian process 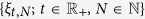 with the non-stationary covariance function


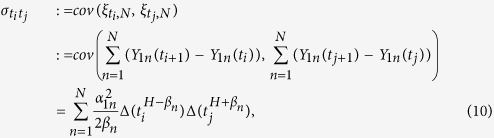


for any *t*_*j*_ < *t*_*i*_ as *N* → ∞.

Let 
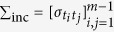
 be the covariance matrix of the increments vector **D** = (Δ*B*_*H*_(*t*_1_), …, Δ*B*_*H*_(*t*_*m*−1_))^T^ and thus the likelihood function is


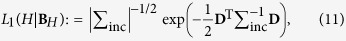


for *H* ∈ Θ_1_. So, the ML estimate of Hurst index, called 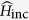, can be obtained by


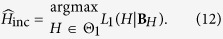


The same approach is employed to calculate the ML estimate of fractal index when the parameter space is Θ_2_ and we denote the induced likelihood function by *L*_2_(*H*|**B**_*H*_).

**Theorem 2.** Suppose that **B**_*H*_ is a sample path of an *f**B**m* with fractal index *H* ∈ Θ_2_. The incremental process {Δ*B*_*H*_(*t*_*i*_), *i* = 1, …, *m* − 1} is the 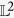 limit of a bi-indexed zero-mean Gaussian process 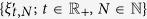 with the non-stationary covariance function as same as the process


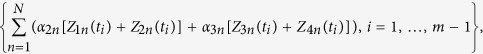


as *N* → ∞ where (*Z*_1*n*_(·), …, *Z*_4*n*_(·)), *n* = 1, …, *N* are independent zero-mean Gaussian random vectors and the covariance elements are given through the equations (22)–(27) in the supplementary file.

Therefore, it suffices to update the elements of 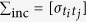 in (11) according to Theorem 2 and consequently


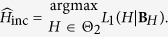


A criticism is the complicating form of the resulting likelihood as a function of *H*. One may claim to compute the exact likelihood using (1). Note that the direct use of (1) yields an in-differentiable function of *H*; while for *H* > 1/2, *β*_*n*_ does not appear in the approximation of the likelihood function. Thus, the target function for maximization belongs to the class of 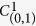 functions and is smooth enough for using numerical methods in computational procedures.

### Bayesian discussion

The approximation of the likelihood function depends on whether *H* ≤ 1/2 or not. According to (1), the fBm is short-range dependent when *H* ∈ Θ_1_ and it becomes a long-range dependent process as well as *H* crosses the threshold 1/2. Thus, the realization of fBm for *H* ≤ 1/2 has a rough graph; while it has a low oscillation with a polynomial trend when *H* ∈ Θ_2_. This visually difference in the short-range and long-range dependent fBm’s may help us in constraining the parameter space to accelerate the calculation of ML estimate or to employ priori information for Bayesian inference. Based on the type of dependence, we constrain the parameter space, Θ, into one of the sub-intervals Θ_1_ or Θ_2_. Consequently, the class of prior distributions is categorized into the set of distributions with support on Θ_1_ or Θ_2_.

The procedure of using a priori information is quite simple. We need a likelihood function as the joint distribution of observations given the unknown parameter *H* and a prior distribution. The likelihood function could be explained in terms of the sample path **B**_*H*_ or its increments, **D**. Simply speaking, one can obtain the likelihood function in terms of **B**_*H*_ by employing Proposition 1 or calculate the likelihood in terms of increments by Theorems 1 and 2. Both methods express the likelihood function for *H* ≤ 1/2 and *H* > 1/2, separately.

**Figure 1:**
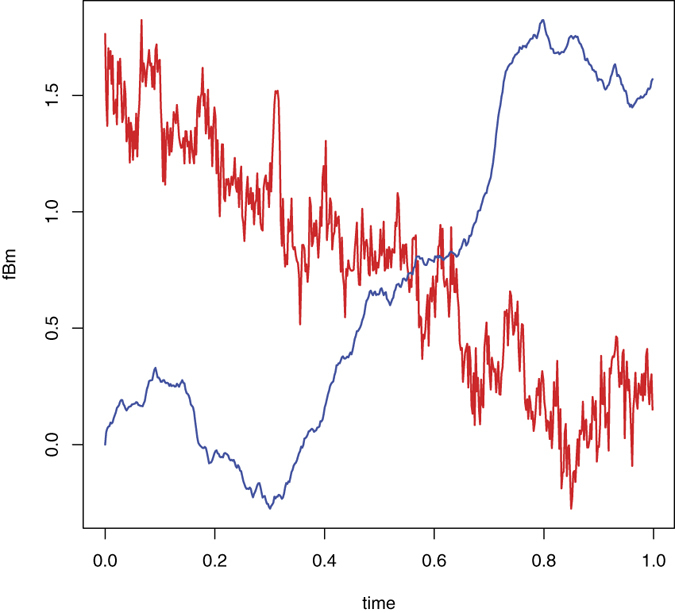
Two realizations of fBm with Hurst indexes *H *=* *1/4 (red line) and *H = *3/4 (blue line). One of the figures are translated through the vertical axis to demonstrate both the realizations in the same Cartesian system.

Let *L*_1_(·|**B**_*H*_) and *L*_2_(·|**B**_*H*_) denote the likelihood functions in terms of **B**_*H*_ for *H* ∈ Θ_1_ and *H* ∈ Θ_2_, respectively (just like the notation which used in (11)) and define *L*_1_(·|**D**) and *L*_2_(·|**D**) in the same way. Let *f*_***D***_(·|*H*) denotes the sample distribution of increments that is rewritten as


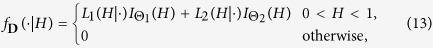


where *I*_*A*_(·) is the indicator function. Although there is no conjugate prior distribution, reducing the parameter space may change the mixture likelihood into the simple multivariate normal. Experts opinions and conjectures on the sample path produce the prior information and may reduce the parameter space. For instance, consider a realization of an fBm with fractal index *H* = 1/4 which the short-range dependence property is very obvious (Fig. 1).

In this case, the expert opinion hopefully suggests to consider *H* in the interval (0, 1/2] and so the second term in (13) is omitted. According to the bounded parameter space, we deduce the use of 1 − 1/2 Beta (*a, b*) and 1/2 Beta (*a, b*) as a priori distributions for long and short-range dependent sample paths, respectively. When the determination of the range is not possible by visual evidences, using a prior distribution on (0, 1) inevitably yields to working with the mixture form of (13). In this situation, we use the Beta (*a, b*) distribution as the prior. Moreover, the suggested priors could be replaced by the uniform distribution as a non-informative prior. The rest is using good candidates for the proposal distributions and performing the MCMC algorithm to report the posteriori issues. The implementation and numerical results of the Bayesian approach are discussed in the following section.

## Results

We separate this section into three main parts. First, the numerical behavior of the likelihood based estimator is studied and then we explain the procedure of Bayesian approach. The third part contains the computation of estimators for a real data.

### Likelihood computation

The consistency of the estimators strongly depends on the mean squared error (MSE) of the approximation of fBm. The structure of the two dimensional Brownian motions in the case *H* > 1/2 could be taken into account to effectively express the terms of series representation. Although the sample path is smoother in the case *H* > 1/2, the MSE of the approximation of the fBm is greater in comparison with the case *H* ≤ 1/2. There is another approximation for fBm that is more effective^4^ than (5) when *H* ∈ Θ_2_. However, it is difficult to obtain a diffusion type process using that approximation. Therefore, we continue the simulation study with the performed likelihoods in previous sections.

Using the circulant embedding method^19^, we first generate the sample path **b** = (*b*_1_, …, *b*_*m*_)^*T*^ of size *m* = 256 from an fBm with known index *H*. We only implement the simulation study for regular observations. The likelihood function given the observed sample path is


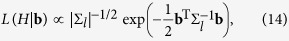


where 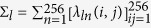, when the generation is implemented according to *H* ∈ Θ_*l*_, *l* = 1, 2. The use of well-known optimization algorithms becomes very difficult due to the complicating form of the likelihood as a function of *H*. The solutions of equation system (8) for a sample path with *H* = 0.1 and *p* = 4 are located at the vector of estimators 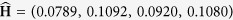 with respect to 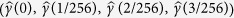. It can be seen that the estimators based on 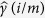 become more precise for larger values of *i*. In one hand, the replication of this simulation study shows less bias but greater variance for greater values of *i*. On the other hand, we found less variance but greater bias for estimators based on 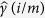 when *i* is small. This is the basis of using the weighted estimator (9). Although one may use the cross validation method to construct a better weight function, for this simulation study we partition off the vector 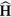 into two vectors with *p*/2 elements and the following weight function is considered for this simulation study


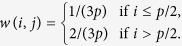


**Figure 2:**
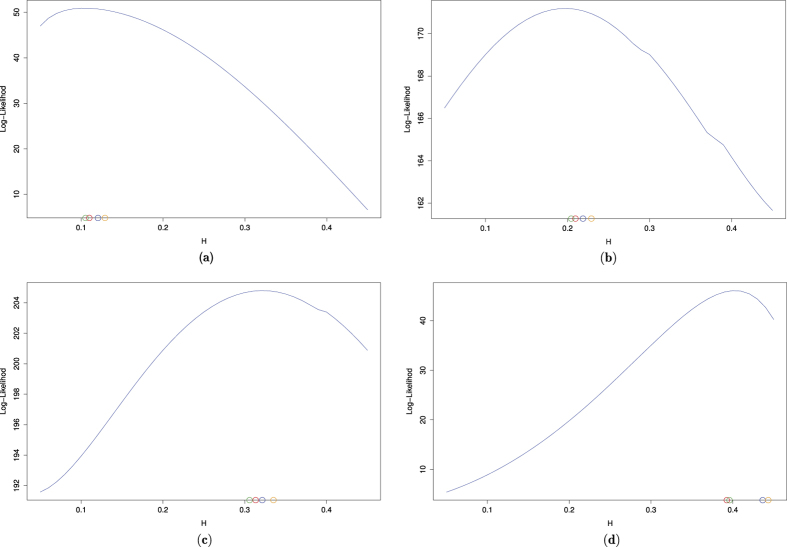
The logarithm of the worst approximated likelihood function based on the incremental approach for sample paths generated with *H* *=* 0.1 (top left, **a**), *H* = *0.*2 (top right, **b**), *H* = *0.*3 (bottom left, **c**) and *H* = *0.*4, (bottom right, **d**). The plots also demonstrate the resulting 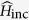 (green circle,) for the generated sample path along the resulting 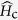
's for *p* = 4 (red circle,), 8 (blue circle,), and 16 (orange circle,).

The second column of Table 1 shows the MSE of 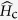 when the unknown parameter *H* is attributed to one of the intervals (0, 1/2] or (1/2, 1). Each reported MSE in this paper is computed by replication of estimation procedure for 100 times. Figure 2 shows the position of the ML estimator and 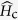 based on the autocovariance estimation at lags 0, …, *p* − 1 for *p* = 4, 8, and 16. Obviously, 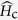 becomes closer to the ML estimate by decreasing *p*. Although the presented estimators 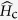 defined by (9) are not ML estimates, they are near to the ML estimates computed from the approximated likelihood function of the increments. Moreover, 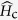 numerically dominates all the simple ML estimates 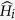 in (9) for *p* = 4.

To implement the incremental approach, construct the sample increments **d** = (*d*_1_, …, *d*_256_)^*T*^ where *d*_*j*_ = *b*_*j*_ − *b*_*j*−1_, for *j* = 1, …, 257. The likelihood function of the observed increments is then


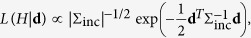


where 
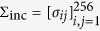
 is computed according to (10) when the generated **b** is a sample path of fBm of index 0 < *H* ≤ 1/2. Fortunately, the smoothing role of differentiation operator makes it possible to maximize the likelihood by employing the simple Particle swarm optimization (PSO) via R package psoptim. The implementation of this procedure for 1/2 < *H* < 1 is not as simple as before. For convenience, let us denote the right hand sides of (A.5)–(A.8) in the supplementary file by *A*_5_(*t, s*) − *A*_8_(*t, s*), and the right hands of (A.9) and (A.10) by *A*_9_(*t*) and *A*_10_(*t*), respectively. According to the Theorem 2, the sub-diagonal elements *σ*_*ij*_, *j* < *i* for 1/2 < *H* < 1 is obtained by


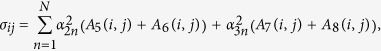


for *i* = 2, …, 256, *j* = 1, …, *i* − 1 and the top-diagonal components are computed using the symmetric behavior of Σ_i*nc*_. Furthermore, the diagonal elements, *σ*_*ii*_, are computed as follows:


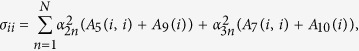


for *i* = 1, …, 256. The covariance matrix Σ_inc_ is now acquired and the nlminb could find the maximum of likelihood function, numerically. The results of this procedure is shown in the 3rd column of Table 1.

In the procedure mentioned above we have known that *H* belongs to Θ_1_ or Θ_2_. When there is no such information about the parameter, the likelihood function compensates this uncertainty. Let us explain this part for 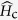. The likelihood function for this problem is


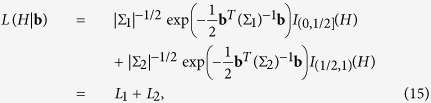


where 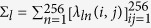, for *l* = 1, 2. Thus, 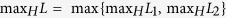 that is equivalent to the maximization of *L*_1_ and *L*_2_ separately and then we pick out the one with the greater value. In this way, we allocate the unknown parameter to one of the intervals along the estimation procedure. We can simply modify this method for the incremental approach by replacing Σ_*l*_ by 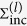, that is the covariance matrix of increments when *H* ∈ Θ_*l*_ for *l* = 1, 2. The results are gathered in the second and third columns of Table 2 and in comparison with Table 1, the MSE of estimators are greater and this shows the effect of more precise parameter spaces used in the previous simulation study.

### Bayesian computation

The Bayesian computation begins with the multiplication of the likelihood function by the prior distribution. Using the mentioned method in 1, the likelihood function computation is straightforward. Remaining is the selection of prior distribution and then the posterior generation using MCMC algorithm. Again let **b** as a sample path of fBm and the parameter space is Θ_1_ or Θ_2_. The suggested prior distributions for *H* ∈ Θ_1_ and *H* ∈ Θ_2_ are respectively 1/2 Beta (*a, a*) and 1 − 1/2 Beta (*a, a*) which both return non-informative uniform prior when *a* = 1. Therefore, the posterior distribution for the parameter space Θ_*l*_, *l* = 1, 2, satisfies


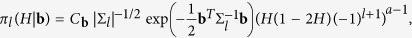


where Σ_*l*_ is the one used in (14) and *C*_**b**_ > 0 is a constant depends on **b**. The posterior distribution has no closed form and inevitably we need to capture the posterior properties using the sample generated by the Metropolis-Hastings algorithm. We use the truncated normal distribution with small enough variance as the proposal density function. More precisely, when the algorithm is in the *j*th iteration, the sample *H*_*j*_ is generated from a normal distribution with mean *H*_*j*−1_ and variance 1/64 which is truncated at points zero and 1/2 when the parameter space is Θ_1_ or truncated at 1/2 and one when the true parameter belongs to Θ_2_. The variance (1/8)^2^ is chosen to cover an interval of length 1/2 by the 95 percent of the values that lie in a band of length 4 × 1/8, according to the famous thumb rule of 68 − 95 − 99.7. Let us denote the density of this distribution by *q*_*l*_(*H*_*j*_|*H*_*j*−1_) where the subscript *l* = 1, 2 represents the parameter space. We accept the generated *H*_*j*_ as a sample of the posterior distribution *π*_*l*_(·|**b**) with probability 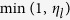 where


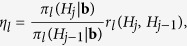


and


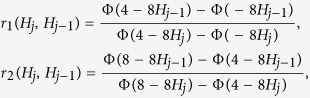


where Φ is the cumulative distribution function of a standard normal random variable. To obtain the true random sample we eliminate the first 2500 generations and for each parameter setting we generate a sample of size 10000 from the corresponding posterior distribution. The results for *a* = 1 and 3 are gathered in the last two columns of Table 1.

Bayesian computation for the general parameter space Θ = (0, 1) is very similar. In this case, we use Beta(*a*_1_, *a*_2_) as the prior distribution. When the observed sample path, **b**, shows an obvious short range dependence behavior, then let *a*_1_ < *a*_2_ and for long range dependence employ *a*_2_ < *a*_1_. Using the notation of (15), the posterior is


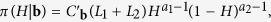


where 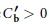 is the normalizing constant. The proposal density *q*(·|*H*_*j*−1_) is a normal distribution with mean *H*_*j*−1_ and variance 1/16 which is truncated at zero and one. Thus, to obtain the *j*th observation from the posterior, generate an observation from density *q*(·|*H*_*j*−1_) and accept it with probability 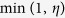 where


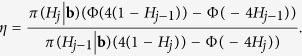


Random samples of size 10000 by considering the burning point at 2500 are used to estimate the Hurst index *H* as the posterior mean and the results are shown in Table 2. The results for *H* = 0.45 and *H* = 0.55 were obtained by use of Beta distribution with parameters *a*_1_ = *a*_2_ = 3 as the prior distribution.

Both the Tables 1 and 2 show that the Bayesian estimator with informative prior returns better results particularly for short range dependent sample paths. In Table 1 and in comparison with Table 2, the MSE of estimators are smaller and this shows the effect of more precise parameter spaces. The error of decomposition and hence the risks of 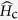 and 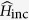 are decreasing in *H*. The fourth column of Table 2 represents the importance of visual information because it returns smaller MSEs in comparison with the last column. Concerning Table 1, to make the range-preserving property for ML estimates, whenever the values of the estimators were out of the parameter spaces, the results were reset to the nearest endpoint. Surprisingly, except for the cases *H* = 0.45 and 0.55, there was no need to reset the values. We also note that the 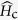 and 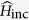 have under and over estimation properties, respectively. For each setting, the trimmed mean base estimato^15^ r, is computed for further comparison. The simple comparison returns the validity of 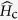, 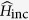 and the Bayesian estimators. Although the computation of Bayes estimators with Beta and Beta type priors are time-consuming, they might compete with other estimators when the lower MSE is the matter.

### Practical aspects

**Figure 3:**
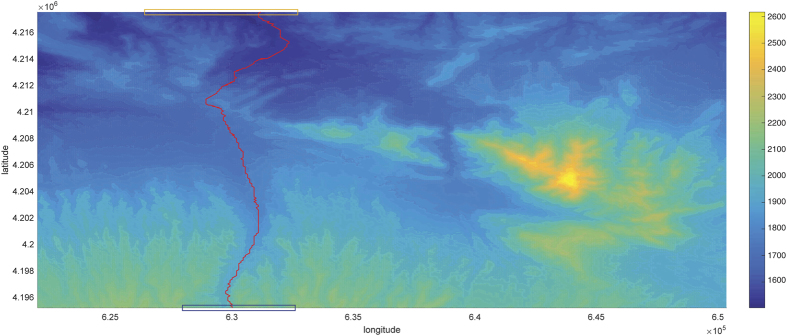
The image of the mountainous area from the Northwest of Iran. The smoothest path between two regions demonstrated by two rectangular in North and South of the region is shown with a red solid line. This path is determined using the Bayesian approach with beta-type prior.

According to our real data, we are confronted with the same situation in the practical problem introduced in the section entitled Practical motivation. Firstly, note that a topographic map of region *S* is a collection of heights {*Z*(*s, t*)} at geographic coordinates (*s, t*) ∈ *S*. Typically, the coordinates are considered as nodes of a regular lattice. Thus, a route begins from a beginning point and is made by connecting some neighboring nodes to achieve the destination node. Precise topographic maps are often considered as fractal surfaces^5^ and estimating the Hurst index is the most interesting problem for such data. We want to construct a road to move across the region *S* (from the North to the South or vice versa) and the smoothness of the road is the characteristic of interest. Figure 3 shows the aerial image of *S* that is a mountainous area from the Northwest of Iran restricted to a rectangle with two opposite vertices located at the geographic *xy* coordinates (621995.073, 4195182.082) and (650345.073, 4217502.082). The image is gridded into a 249 × 316 lattice. We would like to find the smoothest North-South route between two regions indicated by the orange and blue rectangles. According to the cost of computation: (1) The image is rescaled into a 83 × 158 lattice; (2) we restrict our study in the new lattice to those routes made by 260 nodes at most. The *χ*^2^ goodness of fit test of fBm is implemented on the resulting route to insure the assumption of fractional behavior. We refer to ref. 20 for a list of goodness of fit tests including the *χ*^2^ method.

**Figure 4:**
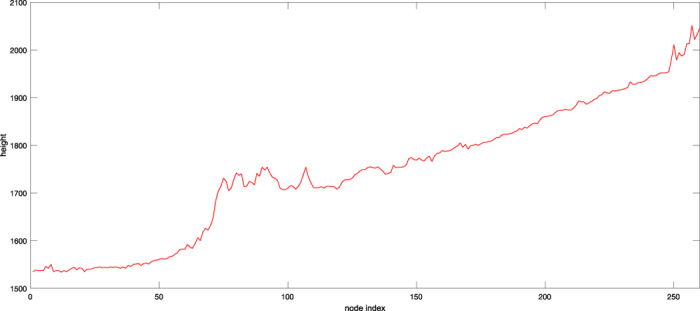
The line transect of the smoothest route detected by the Bayesian approach.

There is a narrow valley at the points with the geographic longitude equal to 6.305 × 10^5^ and we expect to detect the smoothest path near these coordinates. Each route is a sequence in the form of {*Z*(*t*_*i*_, *s*_*i*_)}_*i*_ such that {(*t*_*i*_, *s*_*i*_)}_*i*_ is a set of connected nodes. We first sort all the possible routes with respect to their total oscillation that is simply the sum of the absolute value |*Z*(*t*_*i*_, *s*_*i*_) − *Z*(*t*_*j*_, *s*_*j*_)| for any two successive nodes (*t*_*i*_, *s*_*i*_) and (*t*_*j*_, *s*_*j*_). Then, we candidate half of the roads with smaller oscillations to find the smoothest path. If the expert idea allows us to attribute the Hurst index of these routes to Θ_1_ or Θ_2_ then we can use the beta-type prior to the estimate *H*; or else, the simple uniform prior on (0, 1) will be employed. In this case, it is suggested to employ a beta-type prior on Θ_1_. A decomposition of *N* = 100 is used to approximate the fBm and hence the likelihood function of each route. The smoothest route is the path with the larger estimated *H*. The smoothest detected path is shown with a solid red line in Fig. 3. This route is constructed by 136 nodes and the line transect of the route is provided in Fig. 4. It is worth mentioning that the estimated *H* parameter of this route is 0.42065 based on the Bayesian computation. The estimators based on the discrete variation, sample quantiles and trimmed mean respectively return the values 0.68479, 0.47103 and 0.42557 for this route.

### Model diagnosis

The main characteristic of the fBm is the subdiffusion that is


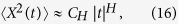


where 0 < *H* < 1, is the Hurst index or subdiffusive exponent and *C*_*H*_ > 0 is diffusion constant. The class of subdiffusion processes contains three well-known stochastic processes; the fBm, continuous-time random walk (CTRW)^21^; and diffusion on the fractal lattice (DFL)^22^,^23^. Although these processes belong to a certain class, their mechanism are rather different^24^. Applying the presented methods to any other process than the fBm will give some *H* which is misleading in the sense that the underlying process is not at all an fBm. In this section we want to test whether the underlying process is an fBm or not. To solve this, we first examine some general properties of the fBm and after obtaining positive results, we use the *p*-variation method^25^ to discriminate the fBm from the CTRW and DFL. The outline of the algorithm is as follows^25^:

- Verifying the stationarity of increments.
- Verifying the normality of the observed path.
- Testing the ergodicity of the increments.
- Testing the *p*-variation properties for some specific *p*’s.
- Testing the filling ratio properties.

We have to apply this procedure to all the possible routes, but for the sake of convenience, we do this on some selected routes, and for the sake of conciseness, we demonstrate the results only for the smoothest detected route.

Let’s start with the first step of the algorithm. The height of each pixel strongly depends on the height of its neighbors. This induces the unit root effect that causes the non-stationarity. A very familiar testing procedure to test the stationarity versus the unit root is KPSS^26^. Using the R package tseries, the KPSS statistic for the increments of the smoothest route is 0.33641 that produces the *p*-value equal to 0.1 and thus the incremental stationarity assumption is verified. Testing the stationarity is not restricted to this method and we refer to ref. 25 for another method that requires more sample paths; whereas we only have one realization and the method can not be implemented here.

Note that there are some reasonable effects that can deflect the trajectory of a subdiffusive process from the Gaussian assumption^27^. That is why we are going to test the normality assumption in the second step. There is plenty of normality testing methods including, Kolmogorov-Smirnov, Anderson-Darling, Shapiro-Wilk, Cramer-von Mises, Shapiro-Francia and Jarque-Bera. There is no certain order for these tests in terms of power, but some of them require the strong assumption of independence of the sample path; which is disaffirmed here under the subdiffusion model. Fortunately, the method of Jarque-Bera is not sensitive to the independence assumption and we employ it to test the normality of increments. We use the R package fBasics and we find out that the *χ*^2^-statistic of Jarque-Bera test is equal to 3.5049 and the corresponding *p*-value is 0.1733 which confirms the normality of the increments of the smoothest path.

Ergodicity states that the long time averages of physical characteristics provide the same information as the corresponding ensemble average. The ergodicity assumption can be violated for sub^28^ and super^29^,^30^ diffusive trajectories even under the power law. It is one of the most important parts of studies, particularly those on single particle tracking. Using the dynamical functional


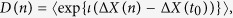


where 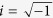, an ergodicity test^31^ is employed here. Set 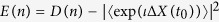. For mixing increments we have^32^ 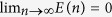 while the increment process is ergodic if and only if the sample mean of 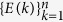 tends to zero, i.e.,


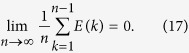


**Figure 5:**
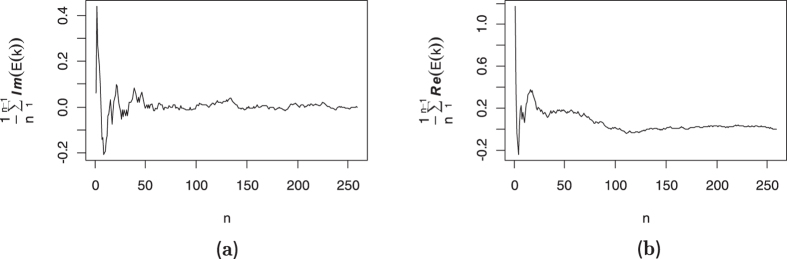
Panel (**a**) shows the imaginary part of the cumulative mean of *E*(*k*) (17) versus *n* while the right panel (**b**) demonstrates the real part. In comparison with the simulated processes analyzed by^31^, the range of both the real and imaginary parts are closer to the zero and figures verifies the ergodicity of the underlying process.

Figure 5 shows the rate of convergence of the cumulative mean to zero. The range of oscillation around zero is very small for the large enough values of *n* that verifies the ergodicity of the smoothest path.

**Figure 6:**
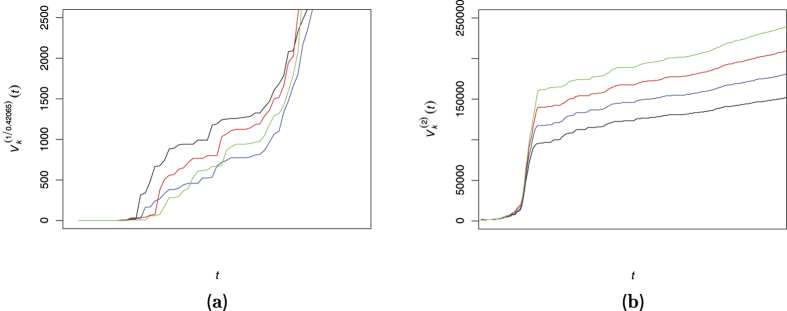
The left panel (**a**) shows the computed 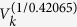 of the smoothest path for *k* = 7 (black line); *k* = 8 (blue line); *k* = 9 (red line) and *k* = 10 (green line). This panel does not demonstrate any trend between *k* and 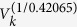. This is the same behavior of any simulated sample path from an fBm of the same index and size. The panel (**b**) shows 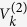 with the same color setting of *k*. It is clear that the 2-variation is increasing in *k* and hence the CTRW model is rejected in favor of fBm.

At this point, the smoothest route passed three steps of the algorithm and in the current step we need to attribute one of the fBm, CTRW or DFL to our data. To this end we use the *p*-variation method^33^ to study the variational behavior of the process. Let 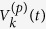 denotes the 1/*p*th root of the incremental *p*-norm, i.e.,


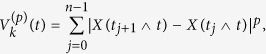


where 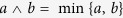. If *X*(*t*) is an fBm then 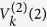 is increasing in *k* and if *X*(*t*) is a CTRW then 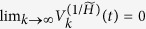 where 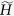 is the estimated Hurst index produced by any proper method. Figure 6 exhibits the resulting values of 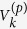 for *p* = 2 and *p* = 1/0.42065. The results verify that the smoothest rout does not follow the CTRW model and thus we remove the CTRW from our list.

**Figure 7:**
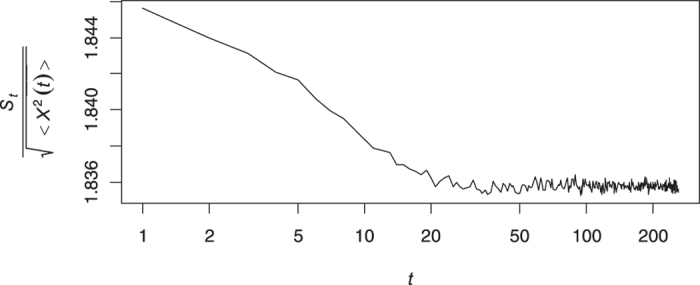
The number of distinct visited sites divided by 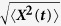 where both the *x* and *y* axes are in logarithmic scale. According to the results, 
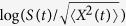
 oscillates smoothly a round a constant value by changing 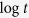.

We have to make a decision between the fBm model and the DFL in the final step. There is a testing procedure based on the mean maximal excursion (MME) method^34^ which needs repeated trajectories from a single process. In some practical problems, the mechanism of data allows gathering independent trajectories of a subdiffusive model with a constant Hurst index^24^. However, in our study, each route is considered as a realization of a subdiffusive process with a corresponding Hurst index. Thus, restricting it to the smoothest route, we only have one trajectory that could be whether an fBm or a DFL. Fortunately, the MME method was modified to be implemented on a single trajectory to decide between the fBm and DFL^35^. Let *S*_*t*_ denotes the total number of distinct visited sites of the process *X*(*t*) up to time *t*. The ratio 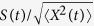 is known as the filling ratio and we have to compute its slope in a log − log scale. The hypothesis of fBm is rejected versus DFL if the slope is far from zero. Figure 7 shows the values of the filling ratios in a double logarithmic scale and the estimated slope is −4.79 × 10^−4^ which implies that the logarithm of the filling ratio and time index are uncorrelated. This means that the dimension of the realization was constructed only by a fBm model and cannot be decomposed into an fBm and a fractal noise. So the DFL model is not verified and the final step in the fBm trajectory diagnosis algorithm is passed.

## Additional Information

**How to cite this article:** Taheriyoun, A. R. and Moghimbeygi, M. Visual information and expert’s idea in Hurst index estimation of the fractional Brownian motion using a diffusion type approximation. *Sci. Rep.* **7**, 42482; doi: 10.1038/srep42482 (2017).

**Publisher's note:** Springer Nature remains neutral with regard to jurisdictional claims in published maps and institutional affiliations.

## Supplementary Material

Supplementary Information

Supplementary Data

## Figures and Tables

**Table 1:** The MSE of the Bayesian estimators, 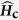 and 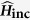 under the restricted parameter space along the the MSE of the estimators based on discrete variation (DV^11^), sample quantiles (SQ^12^) and trimmed mean (TM^15^) under the general parameter space are given.

	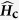	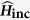	DV	SQ	TM	Bayesian	
						Uniform prior	Beta type prior
*H* = 0.05	13.693	9.633	15.2544	37.7292	18.162	4.566	4.817
0.1	13.631	10.838	17.0344	42.5174	24.017	4.855	4.706
0.2	16.124	11.996	24.8491	47.2209	33.702	4.519	5.440
0.3	18.649	10.488	23.8394	57.5849	28.419	6.259	4.844
0.4	16.653	10.608	34.6774	61.1165	36.690	6.585	4.711
0.45	15.638	9.560	30.3553	55.3652	40.734	4.556	5.387
0.55	14.217	12.635	40.8556	62.8026	42.061	5.356	5.067
0.6	14.612	11.469	38.5361	65.9654	47.568	8.152	5.079
0.7	15.130	11.312	36.7738	55.5099	53.171	8.625	4.785
0.8	14.824	10.607	34.7035	59.9203	42.661	11.408	5.242
0.9	13.980	9.981	30.2872	71.0405	44.464	13.690	4.733
0.95	13.963	10.120	42.3179	59.2273	58.332	12.859	5.004

The results are multiplied by 10^4^.

**Table 2:** The MSE of ML and Bayesian estimators for *H* *∈* (0, 1), multiplied by 10^4^.

	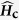	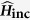	Bayesian	
			skewed beta prior	Uniform prior
*H* = 0.1	42.458	42.983	12.882	36.810
0.3	39.595	36.941	12.512	25.951
0.45	35.937	34.629	5.589	23.242
0.55	37.790	33.498	10.423	18.313
0.7	33.129	32.943	9.435	16.225
0.9	33.876	31.025	5.634	12.320
