# Supplementary material for: Visual information and expert’s idea in Hurst index estimation of the fractional Brownian motion using a diffusion type approximation
Source: Sci Rep. 2017 Feb 14;7:42482. doi: 10.1038/srep42482 (PMC5307349; doi:10.1038/srep42482)
Supplement: Supplementary Information [file srep42482-s1.pdf]

Proofs of theorems for

Visual information and expert's idea in Hurst index estimation of the fractional Brownian motion using a diffusion type approximation

Ali R. Taheriyoun[1] and Meysam Moghimbeygi

Proof of Proposition 1. For given $n \in \mathbb{N}$ and $t > 0$, the summand, $Y_{1n}(t)$, is a non-stationary zero-mean Gaussian process with variance $\lambda_{11}(t)$ where using Itô isometry we have

$$\begin{aligned}\lambda_{11}(t) &= E|Y_{1n}(t)|^2 \\ &= E\Big|\int_{-\infty}^{\log t} \alpha_{1n} t^{H-\beta_n} \mathrm{e}^{\beta_n u} \mathrm{d}B^{(n)}(u)\Big|^2 \\ &= E\int_{-\infty}^{\log t} \alpha_{1n}^2 t^{2(H-\beta_n)} \mathrm{e}^{2u\beta_n} \mathrm{d}u \\ &= \frac{\alpha_{1n}^2 t^{2H}}{2\beta_n} < \infty. \end{aligned} \tag{18}$$

For given n and $t > s$ we deduce that

$$\begin{aligned}\lambda_{12}(t,s) &= E[Y_{1n}(t)Y_{1n}(s)] \\ &= E\Big[\int_{-\infty}^{\log t} \alpha_{1n} t^{H-\beta_n} \mathrm{e}^{\beta_n u} \mathrm{d}B^{(n)}(u) \int_{-\infty}^{\log s} \alpha_{1n} s^{H-\beta_n} \mathrm{e}^{\beta_n u} \mathrm{d}B^{(n)}(u)\Big] \\ &= E\Big[\Big(\int_{-\infty}^{\log s} \alpha_{1n} t^{H-\beta_n} \mathrm{e}^{\beta_n u} \mathrm{d}B^{(n)}(u) + \int_{\log t - \log s}^{\log t} \alpha_{1n} t^{H-\beta_n} \mathrm{e}^{\beta_n u} \mathrm{d}B^{(n)}(u)\Big) \\ &\qquad \int_{-\infty}^{\log s} \alpha_{1n} s^{H-\beta_n} \mathrm{e}^{\beta_n u} \mathrm{d}B^{(n)}(u)\Big] \\ &= E\Big[\int_{-\infty}^{\log s} \alpha_{1n} t^{H-\beta_n} \mathrm{e}^{\beta_n u} \mathrm{d}B^{(n)}(u) \int_{-\infty}^{\log s} \alpha_{1n} s^{H-\beta_n} \mathrm{e}^{\beta_n u} \mathrm{d}B^{(n)}(u)\Big] \\ &= E\Big[\alpha_{1n}^2 t^{H-\beta_n} s^{H-\beta_n} \int_{-\infty}^{\log s} \mathrm{e}^{2u\beta_n} \mathrm{d}u\Big] \\ &= \frac{\alpha_{1n}^2}{2\beta_n} s^{H+\beta_n} t^{H-\beta_n}, \end{aligned}$$

[1](Corresponding author): Department of Statistics, Shahid Beheshti University, Evin, Tehran 1983969411. Email: a_taheriyoun@sbu.ac.ir.

where the fourth equality is due to the independent increment property of the Brownian motion $B^{(n)}(\cdot)$. $\square$

In the sequel, the following lemma is required to prove Theorem 1:

Lemma 0.1. *The incremental process* $\{\Delta B_H(t_i), i=1,\ldots,m-1\}$ *is the limit of a bi-indexed Itô-type process*

$$\left\{\sum_{n=1}^{N}\alpha_{1n}\left[\Delta(t_i^{H-\beta_n})\int_{-\infty}^{\log t_i}\mathrm{e}^{\beta_n s}\mathrm{d}B^{(n)}(s)+t_{i+1}^{H-\beta_n}\int_{\log t_i}^{\log t_{i+1}}\mathrm{e}^{\beta_n s}\mathrm{d}B^{(n)}(s)\right];t\in\mathbb{R}_+,N\in\mathbb{N}\right\},$$

as $N\to\infty$.

Proof. Recall the fact that is the convergences $X_n\xrightarrow{\mathbb{L}^2}X$ and $Y_n\xrightarrow{\mathbb{L}^2}Y$ imply $X_n-Y_n\xrightarrow{\mathbb{L}^2}X-Y$ as $n\to\infty$. Now write the convergence (4b) for t_i and t_{i+1} separately and then using the mentioned fact we have

$$\sum_{n=1}^{N}\alpha_{1n}\left[\int_{-\infty}^{\log t_{i+1}}t_{i+1}^{H-\beta_n}\mathrm{e}^{\beta_n s}\mathrm{d}B^{(n)}(s)-\int_{-\infty}^{\log t_i}t_i^{H-\beta_n}\mathrm{e}^{\beta_n s}\mathrm{d}B^{(n)}(s)\right]\xrightarrow{\mathbb{L}^2}\Delta B_H(t_i). \tag{19}$$

The left hand side of this convergence is simplified into

$$\begin{aligned}
&\sum_{n=1}^{N}\alpha_{1n}\left[t_{i+1}^{H-\beta_n}\int_{-\infty}^{\log t_i}\mathrm{e}^{\beta_n s}\mathrm{d}B^{(n)}(s)+t_{i+1}^{H-\beta_n}\int_{\log t_i}^{\log t_{i+1}}\mathrm{e}^{\beta_n s}\mathrm{d}B^{(n)}(s)\right.\\
-\quad&\left.t_i^{H-\beta_n}\int_{-\infty}^{\log t_i}\mathrm{e}^{\beta_n s}\mathrm{d}B^{(n)}(s)\right]\\
=\quad&\sum_{n=1}^{N}\alpha_{1n}\left[\Delta(t_i^{H-\beta_n})\int_{-\infty}^{\log t_i}\mathrm{e}^{\beta_n s}\mathrm{d}B^{(n)}(s)+t_{i+1}^{H-\beta_n}\int_{\log t_i}^{\log t_{i+1}}\mathrm{e}^{\beta_n s}\mathrm{d}B^{(n)}(s)\right],
\end{aligned}$$

where $\Delta\left(f(t_i)\right)=f(t_{i+1})-f(t_i)$ for any function f and this completes the proof. $\square$

Proof of Theorem 1. The convergence in $\mathbb{L}^2$ implies the convergence of covariances. Therefore, we only need to calculate the covariance function of the left hand side of (19). The first Itô integral in (19) is $\mathcal{F}_{\log t_i}$-measurable and is independent from the second integral.

Employing the Itô isometry for given times $0 < s_i < s_{i+1} \leq t_i < t_{i+1}$ we have

$$
\begin{aligned}
& cov\left(\sum_{n=1}^{N}\alpha_{1n}\left[\Delta(t_i^{H-\beta_n})\int_{-\infty}^{\log t_i}\mathrm{e}^{\beta_n u}\mathrm{d}B^{(n)}(u) + t_{i+1}^{H-\beta_n}\int_{\log t_i}^{\log t_{i+1}}\mathrm{e}^{\beta_n u}\mathrm{d}B^{(n)}(u)\right]\right. \\
& \qquad \left., \sum_{n=1}^{N}\alpha_{1n}\left[\Delta(s_i^{H-\beta_n})\int_{-\infty}^{\log s_i}\mathrm{e}^{\beta_n u}\mathrm{d}B^{(n)}(u) + s_{i+1}^{H-\beta_n}\int_{\log s_i}^{\log s_{i+1}}\mathrm{e}^{\beta_n u}\mathrm{d}B^{(n)}(u)\right]\right) \\
= \; & \sum_{n=1}^{N}\alpha_{1n}^2\left[\Delta(t_i^{H-\beta_n})\Delta(s_i^{H-\beta_n})cov\left(\int_{-\infty}^{\log t_i}\mathrm{e}^{\beta_n u}\mathrm{d}B^{(n)}(u), \int_{-\infty}^{\log s_i}\mathrm{e}^{\beta_n u}\mathrm{d}B^{(n)}(u)\right)\right. \\
+ \; & \Delta(t_i^{H-\beta_n})s_{i+1}^{H-\beta_n}cov\left(\int_{-\infty}^{\log t_i}\mathrm{e}^{\beta_n u}\mathrm{d}B^{(n)}(u), \int_{\log s_i}^{\log s_{i+1}}\mathrm{e}^{\beta_n u}\mathrm{d}B^{(n)}(u)\right) \\
+ \; & \Delta(s_i^{H-\beta_n})t_{i+1}^{H-\beta_n}cov\left(\int_{\log t_i}^{\log t_{i+1}}\mathrm{e}^{\beta_n u}\mathrm{d}B^{(n)}(u), \int_{-\infty}^{\log s_i}\mathrm{e}^{\beta_n u}\mathrm{d}B^{(n)}(u)\right) \\
+ \; & \left. + t_{i+1}^{H-\beta_n}s_{i+1}^{H-\beta_n}cov\left(\int_{\log t_i}^{\log t_{i+1}}\mathrm{e}^{\beta_n u}\mathrm{d}B^{(n)}(u), \int_{\log s_i}^{\log s_{i+1}}\mathrm{e}^{\beta_n u}\mathrm{d}B^{(n)}(u)\right)\right] \\
= \; & \sum_{n=1}^{N}\alpha_{1n}^2\left[\Delta(t_i^{H-\beta_n})\Delta(s_i^{H-\beta_n})\frac{s_i^{2\beta_n}}{2\beta_n} + \Delta(t_i^{H-\beta_n})s_{i+1}^{H-\beta_n}\frac{\Delta(s_i^{2\beta_n})}{2\beta_n} + 0 + 0\right] \\
= \; & \sum_{n=1}^{N}\frac{\alpha_{1n}^2}{2\beta_n}\Delta(t_i^{H-\beta_n})\Delta(s_i^{H+\beta_n}). \qquad (20)
\end{aligned}
$$

□

Proof of Theorem 2. The method is similar to the case $H \leq 1/2$. We therefore sketch here the technique of Lemma 0.1 and Theorem 1 together. Using (4b), we have the convergence

$$
\begin{aligned}
& \sum_{n=1}^{N}\left(\alpha_{2n}\left[\int_{-\infty}^{\log t_{i+1}}\left(\mathrm{e}^{Hs} - t_{i+1}^{H(1-\gamma_{2n})}\mathrm{e}^{\gamma_{2n}Hs}\right)\mathrm{d}B^{(n)}(s)\right.\right. \\
- \; & \left.\int_{-\infty}^{\log t_i}\left(\mathrm{e}^{Hs} - t_i^{H(1-\gamma_{2n})}\mathrm{e}^{\gamma_{2n}Hs}\right)\mathrm{d}B^{(n)}(s)\right] \\
+ \; & \alpha_{3n}\left[\int_{-\infty}^{\log t_{i+1}}\left(t_{i+1}^{2H-1}\mathrm{e}^{(1-H)s} - t_{i+1}^{H-(1-H)\gamma_{3n}}\mathrm{e}^{\gamma_{3n}(1-H)s}\right)\mathrm{d}B'^{(n)}(s)\right. \\
- \; & \left.\left.\int_{-\infty}^{\log t_i}\left(t_i^{2H-1}\mathrm{e}^{(1-H)s} - t_i^{H-(1-H)\gamma_{3n}}\mathrm{e}^{\gamma_{3n}(1-H)s}\right)\mathrm{d}B'^{(n)}(s)\right]\right) \xrightarrow{\mathbb{L}^2} \Delta B_H(t_i),
\end{aligned}
$$

as $N \to \infty$ for each t_i, $i = 1, \ldots, m-1$. We then break those integrals with bounds $-\infty$ to $\log t_{i+1}$ into two integrals over the intervals $(-\infty, \log t_i]$ and $(\log t_i, \log t_{i+1}]$. With the same

terminology of Lemma 0.1, we can write

$$\begin{aligned}
& \sum_{n=1}^{N}\Bigg(\alpha_{2n}\Bigg[-\Delta(t_i^{H(1-\gamma_{2n})})\int_{-\infty}^{\log t_i}\mathrm{e}^{\gamma_{2n}Hs}\mathrm{d}B^{(n)}(s)\\
+ & \int_{\log t_i}^{\log t_{i+1}}\Big(\mathrm{e}^{Hs}-t_{i+1}^{H(1-\gamma_{2n})}\mathrm{e}^{\gamma_{2n}Hs}\Big)\mathrm{d}B^{(n)}(s)\Bigg]\\
+ & \alpha_{3n}\Bigg[\int_{-\infty}^{\log t_i}\Big(-\Delta(t_i^{2H-1})\mathrm{e}^{(1-H)s}-\Delta(t_i^{H-(1-H)\gamma_{3n}})\mathrm{e}^{\gamma_{3n}(1-H)s}\Big)\mathrm{d}B'^{(n)}(s)\\
+ & \int_{\log t_i}^{\log t_{i+1}}\Big(t_{i+1}^{2H-1}\mathrm{e}^{(1-H)s}-t_{i+1}^{H-(1-H)\gamma_{3n}}\mathrm{e}^{\gamma_{3n}(1-H)s}\Big)\mathrm{d}B'^{(n)}(s)\Bigg]\Bigg)\\
\equiv & \sum_{n=1}^{N}\Big(\alpha_{2n}\left[Z_{1n}(t_i)+Z_{2n}(t_i)\right]+\alpha_{3n}\left[Z_{3n}(t_i)+Z_{4n}(t_i)\right]\Big)\\
\xrightarrow{\mathbb{L}^2} & \Delta B_H(t_i).
\end{aligned}$$

The random variables $Z_{1n}(t_i),\ldots,Z_{4n}(t_i)$ are independent since $Z_{1n}(t_i)$ and $Z_{3n}(t_i)$ are $\mathcal{F}_{\log t_i}$-measurable and are independent of $Z_{2n}(t_i)$ and $Z_{4n}(t_i)$. Also, $Z_{1n}(t_i)$ and $Z_{2n}(t_i)$ are defined with respect to the Brownian motion $B^{(n)}$ that is independent of $B'^{(n)}$ and thus

$$\begin{aligned}
\sigma_{t_is_i} &= cov\Bigg(\sum_{n=1}^{N}\Big(\alpha_{2n}\Big[Z_{1n}(t_i)+Z_{2n}(t_i)\Big]+\alpha_{3n}\Big[Z_{3n}(t_i)+Z_{4n}(t_i)\Big]\Big),\\
&\qquad \sum_{n=1}^{N}\Big(\alpha_{2n}\Big[Z_{1n}(s_i)+Z_{2n}(s_i)\Big]+\alpha_{3n}\Big[Z_{3n}(s_i)+Z_{4n}(s_i)\Big]\Big)\Bigg)\\
&= \sum_{n=1}^{N}\alpha_{2n}^2\Big[cov(Z_{1n}(t_i),Z_{1n}(s_i))+cov(Z_{1n}(t_i),Z_{2n}(s_i))\Big] \qquad (21)\\
&+ \sum_{n=1}^{N}\alpha_{3n}^2\Big[cov(Z_{3n}(t_i),Z_{3n}(s_i))+cov(Z_{3n}(t_i),Z_{4n}(s_i))\Big].
\end{aligned}$$

We employ the Itô isometry to calculate the covariances as follows:

$$\begin{aligned} cov(Z_{1n}(t_i), Z_{1n}(s_i)) &= \Delta(t_i^{H(1-\gamma_{2n})})\Delta(s_i^{H(1-\gamma_{2n})}) \\ &\quad \times E\left[\int_{-\infty}^{\log t_i} \mathrm{e}^{\gamma_{2n}Hu}\mathrm{d}B^{(n)}(u)\int_{-\infty}^{\log s_i} \mathrm{e}^{\gamma_{2n}Hu}\mathrm{d}B^{(n)}(u)\right] \\ &= \Delta(t_i^{H(1-\gamma_{2n})})\Delta(s_i^{H(1-\gamma_{2n})})E\left[\left(\int_{-\infty}^{\log s_i} + \int_{\log s_i}^{\log t_i}\right)\int_{-\infty}^{\log s_i}\right] \\ &= \Delta(t_i^{H(1-\gamma_{2n})})\Delta(s_i^{H(1-\gamma_{2n})})E\left[\int_{-\infty}^{\log s_i} \mathrm{e}^{2\gamma_{2n}Hu}\mathrm{d}u\right] \\ &= \Delta(t_i^{H(1-\gamma_{2n})})\Delta(s_i^{H(1-\gamma_{2n})})\frac{2\gamma_{2n}H}{s_i^{2\gamma_{2n}H}}, \end{aligned} \tag{22}$$

and using the same method in breaking the stochastic integrals into the proper sub-intervals we have

$$cov(Z_{1n}(t_i), Z_{2n}(s_i)) = \Delta(t_i^{H(1-\gamma_{2n})})\left[\frac{\Delta(s_i^{H(\gamma_{2n}+1)})}{H(\gamma_{2n}+1)} - \frac{s_{i+1}^{H(1-\gamma_{2n})}\Delta(s_i^{2\gamma_{2n}H})}{2\gamma_{2n}H}\right], \tag{23}$$

$$\begin{aligned} cov(Z_{3n}(t_i), Z_{3n}(s_i)) &= \Delta(t_i^{2H-1})\Delta(s_i^{2H-1})\frac{s_i^{2(1-H)}}{2(1-H)} \\ &+ \left(\Delta(t_i^{2H-1})\Delta(s_i^{H-(1-H)\gamma_{3n}}) + \Delta(t_i^{H-(1-H)\gamma_{3n}})\Delta(s_i^{2H-1})\right)\frac{s_i^{(1-H)(1+\gamma_{3n})}}{(1-H)(1+\gamma_{3n})} \\ &+ \Delta(t_i^{H-(1-H)\gamma_{3n}})\Delta(s_i^{H-(1-H)\gamma_{3n}})\frac{s_i^{2(1-H)\gamma_{3n}}}{2(1-H)\gamma_{3n}}, \end{aligned} \tag{24}$$

and

$$\begin{aligned} cov(Z_{3n}(t_i), Z_{4n}(s_i)) &= -\Delta(t_i^{2H-1})s_{i+1}^{2H-1}\Delta(s_i^{2(1-H)})r \\ &+ \left(\Delta(t_i^{2H-1})s_{i+1}^{H-(1_H)\gamma_{3n}} - \Delta(t_i^{H-(1-H)\gamma_{3n}})s_{i+1}^{2H-1}\right)\Delta(s_i^{(1-H)(1+\gamma_{3n})}) \\ &+ \Delta(t_i^{H-(1-H)\gamma_{3n}})s_{i+1}^{H-(1-H)\gamma_{3n}})\Delta(s_i^{2(1-H)\gamma_{3n}}). \end{aligned} \tag{25}$$

Note that for the diagonal elements, $\sigma_{t_it_i}$, we have

$$\begin{aligned} \sigma_{t_it_i} &= cov\Bigg(\sum_{n=1}^{N}\Big(\alpha_{2n}\Big[Z_{1n}(t_i) + Z_{2n}(t_i)\Big] + \alpha_{3n}\Big[Z_{3n}(t_i) + Z_{4n}(t_i)\Big]\Big), \\ &\qquad \sum_{n=1}^{N}\Big(\alpha_{2n}\Big[Z_{1n}(t_i) + Z_{2n}(t_i)\Big] + \alpha_{3n}\Big[Z_{3n}(t_i) + Z_{4n}(t_i)\Big]\Big)\Bigg) \\ &= \sum_{n=1}^{N}\left\{\alpha_{2n}^2\left(E[Z_{1n}(t_i)]^2 + E[Z_{2n}(t_i)]^2\right) + \alpha_{3n}^2\left(E[Z_{3n}(t_i)]^2 + E[Z_{4n}(t_i)]^2\right)\right\}, \end{aligned}$$

since $E[Z_{1n}Z_{3n}] = E[Z_{2n}Z_{4n}] = 0$. Thus, it remains to compute $E[Z_{2n}]^2$, $E[Z_{4n}]^2$, where using the same method mentioned above, we have

$$\begin{aligned} E[Z_{2n}]^2 &= \frac{1}{H}\left(\frac{1}{2}\Delta\left(t_i^{2H}\right) + t_{i+1}^{2H}\left(\frac{1-3\gamma_{2n}}{2\gamma_{2n}(1+\gamma_{2n})}\right) - \frac{1}{2\gamma_{2n}}t_{i+1}^{2H(1-\gamma_{2n})}t_i^{2\gamma_{2n}H}\right. \qquad (26) \\ &+ \left.\frac{2}{1+\gamma_{2n}}t_{i+1}^{H(1-\gamma_{2n})}t_i^{\gamma_{2n}H}\right), \\ E[Z_{4n}]^2 &= 2t_{i+1}^{3H-\gamma_{3n}(1_H)-1}t_i^{(1+\gamma_{3n})(1-H)} - t_{i+1}^{2H-2\gamma_{3n}(1-H)}t_i^{2\gamma_{3n}(1_H)} \qquad (27) \\ &- t_{i+1}^{2(2H-1)}t_i^{2(1-H)}. \end{aligned}$$

□
